# Impact of Job Demands on Employee Learning: The Moderating Role of Human–Machine Cooperation Relationship

**DOI:** 10.1155/2022/7406716

**Published:** 2022-12-06

**Authors:** Wang Sen, Zhu Xiaomei, Deng Lin

**Affiliations:** ^1^School of Management, Beijing Union University, Beijing, China; ^2^Lubar School of Business, University of Wisconsin-Milwaukee, Milwaukee, WI, USA

## Abstract

New artificial intelligence (AI) technologies are applied to work scenarios, which may change job demands and affect employees' learning. Based on the resource conservation theory, the impact of job demands on employee learning was evaluated in the context of AI. The study further explores the moderating effect of the human–machine cooperation relationship between them. By collecting 500 valid questionnaires, a hierarchical regression for the test was performed. Results indicate that, in the AI application scenario, a *U*-shaped relationship exists between job demands and employee learning. Second, the human–machine cooperation relationship moderates the *U*-shaped curvilinear relationship between job demands and employees' learning. In this study, AI is introduced into the field of employee psychology and behavior, enriching the research into the relationship between job demands and employee learning.

## 1. Introduction

Nowadays, artificial intelligence (AI) is rapidly empowering traditional industries. For example, speech recognition, driverless cars, machine translation, and industrial robots are all widely applied in service, manufacturing, and other industries, thereby changing job demands for employees [1]. Jobs that are highly repetitive and easily simulated by artificial machines are being replaced, and employees are taking on more creative jobs. For instance, a “robot advisor” in the banking industry can automatically adjust financial investment portfolios according to income goals and risk tolerance of customers. Moreover, employees need to provide more humane services and create financial projects with more investment value. Human resource specialists no longer screen complicated resumes of candidates but instead focus more on providing enterprises with flexible and suitable talent training strategies for enterprise development. Changes in job demands pose new challenges to employees. An Oracle survey found that 51% of employees could not adapt to the company's AI development and had negative emotional experiences, which reduced their enthusiasm for learning. Therefore, how to make employees actively adapt to the changes in AI job demands and maintain continuous learning is of important practical significance.

### 1.1. Reviews of the Effects of AI on Employee Behavior

The effects of AI on employees have attracted much attention from researchers in various areas, and most existing research focuses on the macroscopic level. On the one hand, these studies highlighted that AI influences the labor force across industries and sectors [2]. Acemoglu and Restrepo [3] found that AI technologies can increase employment demand in nonsmart sectors by boosting overall economic productivity. Dauth et al. [4] noted that introducing AI technology will generate new jobs and absorb employees. On the other hand, recent studies have highlighted the measurement of the employment substitution risk of AI technology. Frey and Osborne [5] pioneered a method for measuring occupational substitutable risk and predicted that 47% of United States-based jobs face a high substitution risk.

Some scholars focus on the effect of AI on employee motivation and satisfaction [6]. Based on the self-determination theory, Arnaud and Chandon [7] found that monitoring systemic extensiveness has a negative effect on employees' intrinsic motivation. Based on social information processing theory, Stanton and Julian [8] discovered that communication with AI fails to convey interpersonal cues. Moreover, employees in the organization develop more task-oriented, instrumental connections [9] than emotional connections [10], which leads to more social undermining [11]. Our study advances this line of research in the context of AI's introduction into firms by determining how job demands influence employee learning.

### 1.2. The Effect of Job Demands on Employee Learning in the Context of AI

With the introduction of AI into enterprises, job demands are characterized by the diversification of cross-border skills, high-level skills, and the complexity of human–computer and interpersonal cooperation skills [12]. According to the job demands resource model (JD-R model), job demands directly change employees' psychological resources [13]. Employees need to establish and adapt emotional and relationship resources for working with intelligent machines. These resources are important sources to stimulate the active learning of individuals [14]. Based on the theory of conservation of resources, individuals will gain or lose resources during their interactions with surrounding environmental elements. Facing the loss of resources, individuals are more inclined to adopt passive, withdrawal-based, and rebellious coping psychology and behavior, whereas acquiring resources makes individuals more inclined to adopt active psychology and behavior [15]. With the introduction of AI into firms, on the one hand, human–machine interaction causes several machines to replace employees' duties. Moreover, the anxiety of “machine substitution” diminishes employees' psychological resources, which is conducive to establishing negative emotions and impairing the formation of relationships [16]. On the other hand, intelligent machines replace employees to complete simple and repetitive tasks [17] and increase employees' work efficiency. Therefore, the establishment of positive emotional relationships between humans and machines is strengthened, and individual learning is promoted. Two diametrically opposed emotions act on employee learning simultaneously, which may lead to a periodic decline or increase in the impact of changes in work requirements on employee learning. This case provides a theoretical basis and a nonlinear perspective for exploring the relationship between job demands and employee learning.

In addition, we attempt to determine the boundary conditions whereby job demands affect employee learning. In the application scenario of AI technology, the continuous interaction between employees and intelligent machines has given birth to the human–machine cooperation relationship between employees and intelligent machines. The human–machine cooperation relationship emphasizes that human beings should interact and cooperate with machines to complete tasks [18, 19]. After the introduction of intelligent machines into enterprises, the human–machine cooperation relationship was formed. The daily tasks, such as repeatability, compliance, and system processing, were more often undertaken by machines [20], and the creative, social, and interpersonal tasks were undertaken by employees [17]. The human–machine cooperation relationship has changed employees' knowledge, emotional, and relationship resources [21] and caused psychological and emotional changes in employees. Therefore, the human–machine cooperation relationship may affect the relationship among job demands, competency needs, and employee learning.

Based on the self-determination theory, this study reveals the mechanism of impact of job demands on employee learning in the application scenario of AI. Specifically, the study examines how changes in job demand affect employee learning in AI application scenarios. Is the indirect effect of job demands on employee learning affected by the human–machine cooperation relationship? By collecting 500 valid questionnaires from 100 AI application enterprises, the results show that job demands have a nonlinear impact on learning. That is, the improvement of job demands initially declines employees' learning and then increases. Thus, the stronger the human–machine cooperation relationship, the more significant the influence of job demands on employee learning.

### 1.3. Contribution

This study aims to contribute theoretically to the following aspects. First, this study estimates the impact of job demands on employees' psychology under the background of AI and expands the research on the relationship between job demands and employee learning. Previous studies were mostly based on the JD-R model to evaluate the impact of job demands on individual psychology [22]. Studies of the relationship between job demands and employee learning are limited. In addition, previous studies found that a linear relationship exists between them in traditional working scenarios [15]. The present study focuses on AI application scenarios and finds that there exists a nonlinear relationship between job demands and employee learning. Second, this study assesses the impact of job demands on employee learning from the perspective of human–machine cooperation. The existing literature expands on the internal and external variables of the organization, such as job remodeling, organizational factors, and family factors, which can affect the relationship between job demands and employee psychology. This study finds that in the AI scene, a new type of interpersonal relationship has been formed between humans and intelligent machines. This kind of human–computer cooperation relationship plays a regulating role between job demands and employee learning, extending the JD-R model to a certain extent.

### 1.4. Structural Arrangements

The remainder of this paper is organized as follows: first, we review the studies involving employee learning and present the underlying mechanisms of the direct effects of the independent variables and the indirect effects of the contingent variables. Second, a research method is developed to collect data and measure the variables analyzed in this study. Third, we provide the results of hypothesis testing. Fourth, the contributions and implications of our research are discussed, and the limitations are identified. Finally, we conclude the study.

## 2. Literature Review and Research Hypotheses

### 2.1. Impact of Job Demands on Employee Learning in AI Scenarios

Employee learning is a process in which individuals use knowledge and skills and feel self-growth [14, 23]. In AI application scenarios, job demands may affect employee learning. The changes in job demands caused by technological changes mainly reflect the higher requirements on the diversity of employees' skills and their cooperation with machinery and equipment [24], which require employees to invest time and energy to complete tasks within a specific period of time and acquire necessary knowledge and skills [25]. However, additional learning will divert employees' work attention, reduce their focus on work tasks, and diminish their interest in exploring existing jobs, thereby weakening employees' enthusiasm for learning [26]. In addition, high job demands usually make employees face job uncertainty [27], producing a serious sense of job insecurity and affecting their psychology and behavior [28]. In the face of the introduction of new AI technology, the job demands of enterprise employees have changed greatly, making employees feel uncertain about their future work and suffer psychological anxiety, decreasing their enthusiasm for learning [29]. However, AI could make people more efficient [30]. The introduction of new AI technologies into enterprises has led to innovative and flexible work characteristics, which have prompted employees to start learning new skills for their own vitality and their continuous learning state [31]. In practice, the impact of changes in job demands caused by AI on employees' learning also differs. The research group examined manufacturing and service-related enterprises. When visiting a communication equipment manufacturing factory, one manager found that UAV inventory checking reduced the work intensity of employees, decreased their work error rate, and made them show high enthusiasm for learning new equipment. During the investigation in the Haidilao smart restaurant and Jijihong restaurant, it was found that the waiters welcomed the food delivery robot, were willing to communicate and cooperate with the smart machine, and were keen on constantly learning new skills to adapt to job-related changes [32]. However, an interview with a senior executive of a communication equipment company found that she was worried about the continuous improvement of job demands and feared that the emergence of AI would replace most workers and lead to mass unemployment. In the application of AI technology, job demands may exert a dual effect of promoting and inhibiting the impact on employees' learning.

At the initial stage of the application of AI, the collaboration between people and intelligent machines is in the running period [33]. The working environment is full of uncertainty and uncontrollability for employees. Employees are full of anxiety and insecurity [34]. Focusing on work tasks is difficult, and they are afraid or unwilling to make new attempts at work. Employees are often exhausted and unable to learn and grow [35]. With the continuous deepening of the application of AI, employees gradually adapt to the state of continuous learning through self-adjustment. They start to focus on work and are interested in exploring work. The cooperation between people and intelligent machines is becoming increasingly tacit [36]. At this time, employees often experience the excitement, sense of achievement, and transcendence brought by the mastery of new technologies in their work. Employees exhibit abundant energy and vitality and grow and develop through continuous learning. Therefore, the following hypothesis is posited:

- H1: There is a *U*-shaped relationship between job demands and employee learning.

### 2.2. Moderating Effect of the Human–Machine Cooperative Relationship

Employee learning is not only derived from individual independent thoughts but also occurs in social interaction [37]. In the process of interaction, organizational context, work resources, and relationship resources will affect employees' learning enthusiasm. AI has thinking and behavioral abilities akin to those of human beings. It has rich knowledge reserves, supercomputing ability, and a human-like expression system [38]. The human–machine cooperation relationship is one of interdependence and cooperation between man and intelligent machines [39, 40]. For example, the restaurant's food delivery robot liberates the food delivery personnel from the heavy physical labor of providing food. The focus of employees' work has shifted to serving customers' personalized needs. With the wide application of AI in the field of work, the cooperation, dependence, and integration between humans and intelligent machines in the field of work are becoming closer [41]. AI can think and act in a manner similar to human beings, with rich knowledge reserves, supercomputing ability, and human-like systems of expression [38]. The rapid processing of procedural and routine work by AI improves work efficiency, frees employees from heavy and tedious work, and enables employees to focus on more core work tasks without interference, actively explore, and seek new solutions [42]. With the cooperation of AI, employees are more liable to feel a sense of support and competence. Therefore, under the same level of job demands, a high-quality human–machine cooperation relationship will bring employees a higher level of employee learning.

At the initial stage of the application of AI technology, the postskill level changes from low to medium. The cooperation between employees and intelligent machines is in the running period. Employees feel that they have lost their sense of control and ability to work, and their level of satisfaction decreases. At this stage, the more employees rely on intelligent machines, the closer the human–machine cooperation relationship [43]. The higher job demands mean that employees need to pay higher costs to readapt to the human–machine cooperation. The higher the energy loss, the more evident the decline in employee learning. When the postskill level changes from medium to high, the cooperation between employees and intelligent machines becomes smoother. Moreover, employees' sense of achievement and ability to work are enhanced, and their competency needs are met [44]. At this stage, the closer the human–machine cooperation relationship, the more employees can obtain the input of knowledge, emotion, and relationship resources in their work and the more evident the rise of employees' learning. Therefore, the following hypothesis is posited:

- H2: The human–machine cooperation relationship positively moderates the *U*-shaped relationship between job demands and employee learning.

**Figure 1:**
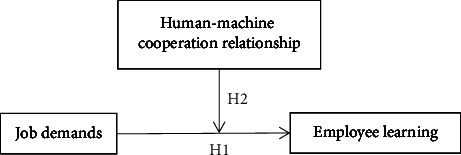
Conceptual model.

Together, these hypotheses lead us to propose the conceptual model shown in Figure 1.

## 3. Methodology

### 3.1. Sample and Data Collection

In our study, the employees from firms that applied AI were selected as research objects. These firms are all from Beijing, China, where many AI firms are gathered. In-depth interviews with three firms applying AI were conducted before undertaking the formal questionnaire-based part of the present research. Moreover, the interview mainly focused on the changes in the work content, psychology, and behavioral perception of employees after the introduction of AI into firms. After the interview, questionnaires were issued to 20 participants as a pilot test. According to feedback, we made detailed revisions to the questionnaire and used the revised version thereafter.

Then, we cooperated with a management consulting firm to help us select suitable firms and issued questionnaires with the assistance of administrative agencies. Moreover, government administrative agencies were responsible for collecting the questionnaires. Experienced interviewers were recruited and trained before conducting the on-site survey. To encourage the participants to keep neutrality and objectivity, we explained to them that this survey was for academic use only, and the confidentiality of the data collected was reassured [15, 45]. Moreover, to protect the participants from any adverse consequences, we carefully crafted the questionnaire with no sensitive information involved, such as firm name, code, location, and contact number [46]. Participants in the survey also need to meet the following criteria: (1) The firms have introduced AI-related equipment or technology. (2) Participants' leaders are willing to cooperate with the survey. (3) Participants keep the contents of the survey confidential and do not inform colleagues.

Furthermore, two versions of questionnaires at different times were distributed to ensure the quality of the collected data. Version A of the questionnaire was filled out by employees, including work demands, human–machine interaction, and other variable-related questions. Two months later, we handed out Version B of the questionnaire to the previous employees and asked them to answer their learning-involved items. After deleting invalid and unmatched questionnaires, 500 available questionnaires were received.

Table 1 shows the demographic profiles of participants in detail, which present the gender, age, and education level. The results indicate that the majority of participants are in the range of 26 to 45 years old. Most participants (70.4%) have a university degree.

### 3.2. Measures

- Job demands. Based on the classic scale form [47], we increased the application background of AI to fit this research topic. Then, the scale was finalized, including “After the introduction of AI technology or equipment, my job skill level is higher” and the other four items.
- Human–machine cooperation relationship. This variable follows a three-step procedure for scale development. For the first step, the method of literature deduction and in-depth interview is used to determine the initial measurement items. The existing relevant studies were reviewed, and human–machine cooperation manifested itself in the cooperative relationship between employees and intelligent machines in the work field [40]. Considering that the work of intelligent machines is highly related to the use of computers, we referred to the scale of the extent of computer use [48] and replaced the use of computers with AI, which indirectly represents the extent of cooperation between humans and machines. The relationship between employees and intelligent machines is considered by the friendship subscale in the work characteristics scale [49], which denotes employees' attitude toward intelligent machines in the human–machine interaction. From the literature review, 20 employees from the three companies that apply AI were interviewed, and the questions on the scale were revised to ensure that they were suitable for human–machine interaction. For the second step, to verify the validity of the questionnaire, exploratory factors were studied to correct the measurement items. The first 100 valid questionnaires were collected for verification. An exploratory factor analysis was conducted using SPSS 22.0. In addition, we used the orthogonal method to rotate and the principal component analysis method simultaneously. Finally, four factors were separated. The factor loadings are all above 0.50, and no significant cross-loading exists. All items could explain the variance of 74.49%. In addition, the value of the KMO test is greater than 0.7, and the value of Cronbach's *α* exceeds 0.6. Third, AMOS was used for a confirmatory factor analysis to verify the scale. The factor loadings of the confirmatory factors are mostly above 0.70, and their comprehensive reliability coefficients and average extraction variances are also greater than the threshold values of 0.70 and 0.50, respectively. This result implies that the scale of the human–machine cooperation relationship demonstrates a good level of reliability. Moreover, the final scale items included four items, such as “Long working hours with AI devices at work” and “I like AI devices at work.”
- Learning. Referring to Porath et al. [50], three statements are included: “I often feel that I am learning,” “I often feel that I am growing,” and “I often feel that I am constantly improving.”

Demographic characteristics, such as gender, age, position, and educational background, may affect employee learning. Hence, they were also included in the questionnaire as control variables for measurement in this study.

### 3.3. Reliability and Validity

The composite reliability is the combination of the reliability of all measured variables, indicating the internal consistency of the facet indicators. The scores of the five variables in this study are all greater than 0.7, suggesting an adequate level of reliability [51]. Cronbach's *α* refers to the average of the split-half reliability coefficients obtained by all possible item division methods of the scale, and it is more sensitive to the number of scale items. The more items, the higher Cronbach's *α* [52]. In our study, the number of variable indicators is less than 6, but the value of Cronbach's *α* coefficient is greater than 0.6. Thus, the scales have high internal consistency [53]. The average variance extracted (AVE) was used to test the convergence validity of structural variables. The AVE score should be greater than 0.5, but 0.36–0.5 is acceptable [54]. The AVE scores of the job demands are all greater than 0.5, indicating that the variables have high convergence validity. Although AVE values of the human–machine cooperation relationship and learning are less than 0.5, but more than 0.36, indicating convergence validity of this variable is acceptable, and factor loadings are acceptable for convergent validity as there are no items with loading below 0.5 [55], as shown in Table 2.

Three-factor models are formed according to the combination of variables.  Table 3 displays the results of the confirmatory factor analysis. The goodness of fit test of the three-factor model is significantly better than that of other competitive models, which can further show that the variables in this study have no significant multicollinearity problems.

### 3.4. Common Method Bias

Considering that the same data sources in this study may result in common method bias, the source of deviation during the design and distribution of the questionnaire was controlled. For example, all the questionnaires were filled out by two types of respondents. The variables were measured from different sources. Anonymous evaluation was employed to reduce the subject's guess about the purpose of the measurement. Furthermore, the Harman single-factor method was adopted to test the common method bias of the scale. Through an exploratory factor analysis, multiple factors were extracted from the test. Moreover, the cumulative variance percentage of the first component was 15.18%, which was less than 50%, indicating that no serious common method deviation exists in this scale [56].

Moreover, a confirmatory factor analysis was performed by selecting a marker variable that is unrelated to our research model and to any studied variables. Then, all studied variables were loaded onto this common method factor, and the result shows that the model fit is poor (CFI = 0.682). Overall, these analyses imply that the common method bias is not a serious concern in the present study.

## 4. Results

### 4.1. Analysis of Descriptive Statistics and Correlation

Table 4 shows the mean and standard deviation of each variable, and the correlation coefficient between variables is also shown. From the results, there is a significant correlation between job demands and learning, and whether the relationship between the two variables exists needs to be further assessed using regression methods.

### 4.2. Analysis of Regression Results between Job Demand and Employee Learning

To compare coefficients concerning the relative explanatory power of the dependent variable in a direct manner, the results of the hierarchical regression estimates were obtained [57]. The results were used to analyze and test the *U*-shaped curve relationship between job requirements and employee learning (Table 5). First, we added job demands and quadratic components of job demands to M1 and M2. M1 shows that, compared with females, male employees are more enthusiastic about learning at work. With increasing age, employee enthusiasm for learning declines (albeit not to any significant extent). Thus, the correlation between the two variables is not high. Employees with higher education and a higher position prefer to learn to a greater extent. Most importantly, the effect of job demands on employee learning is not significant (*β* = 0.134, *p* > 0.1). In M2, after controlling for gender, age, position, and educational background, the first-order coefficient of job requirements for learning is significantly negative (*β* = −0.583, *p* < 0.001), and the second-order coefficient is significantly positive (*β* = 0.101, *p* < 0.001). Δ*R*^2^ is 0.029, with a significant result, indicating that there is a *U*-shaped relationship between job requirements and learning; thus, H1 is supported.

### 4.3. Analysis of Moderating Effect Results

In model 3, we added the interaction item of job demands and the human–machine cooperation relationship and the interaction item of the job demands square and the human–machine cooperation relationship in model 4. From the results, the interaction effect is found to be significant (*β* = 0.095, *p* < 0.05), which mainly shows that the human–machine cooperation relationship plays a moderating effect in the *U*-shaped relationship between job demands and employee learning. Subsequently, we made a simple estimation of the curve slope, with the mean value of the human–machine cooperation relationship plus or minus one standard deviation [58]. The results show that, under the high human–machine cooperation relationship (*β* = 0.019, *p* < 0.001) and a low human–machine cooperation relationship (*β* = 0.013, *p* < 0.05) (Table 6), the relationship between job demands and employee learning is quadratic. Thus, H2 is verified.

**Figure 2:**
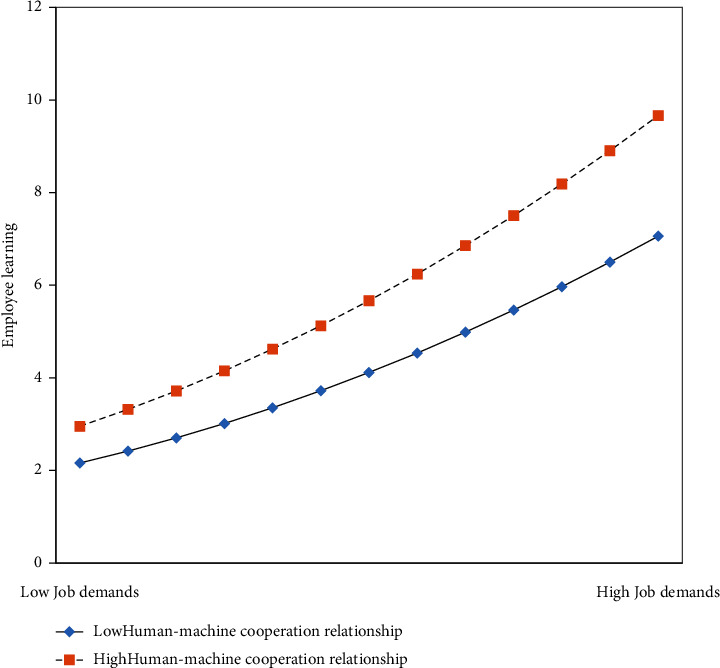
Moderating effect of the human–machine cooperation relationship on job demands and employee learning.

Furthermore, the influences of high and low levels of the human–machine cooperation relationship on the relationships between job demands and employee learning were studied, as plotted in Figure 2, thereby evaluating the moderating effect [59]. As shown in Figure 2, at high levels of human–machine cooperation relationship, the steeper slope of job demands suggests a positive effect on employee learning, supporting H2.

## 5. Discussion

This study reveals the mechanism of the influence of job demands on employees' learning under AI application scenarios. We find that after the implementation of AI technology or equipment in enterprises, with the improvement of job demands, employee learning initially decreases and then increases. The human–machine cooperation relationship enhances the nonlinear relationship between job demands and employee learning.

### 5.1. Theoretical Contributions

By revealing the mechanism of job demands on employee learning under AI application scenarios, this study shows two theoretical contributions.

#### 5.1.1. Contribution to Employee Learning Research

First, AI is introduced into the field of organizational behavior and a *U*-shaped relationship is found between job demands and employee learning. Different from the conclusion obtained by Prem et al. [31] that a linear relationship exists between job demands and employee learning, we find that in the context of AI application, a *U*-shaped relationship exists between job demands and employee learning. Faced with the changes in job demands brought by AI, employee psychology changes from initial resistance to gradual understanding, recognition, and acceptance, resulting in a *U*-shaped relationship between job demands and employee learning. The results of this study break through the conclusion that a linear relationship exists between job demands and employee learning [60] and expand the research on employee psychology and behavior under the background of AI.

#### 5.1.2. Contribution to Human–Machine Cooperation Relationship Research

Second, the human–machine cooperation relationship exerts a moderate effect on the relationship between job demands and employee learning. In addition to the organizational situation and self-cognition, individual learning is also affected by peer relationships [61]. In the context of AI, the human–machine cooperation relationship is a new human-like relationship formed between humans and intelligent machines [62]. In the process of human–machine interaction, intelligent machines lead to changes in the knowledge resources [40, 63], including positive meaning resources and emotional resources owned by employees, which changes employees' working attitude and behavior [64]. This finding suggests that the human–machine cooperation relationship may affect the relationship between job demands and employee competency requirements. However, the correlation between humans and intelligent machines has not been considered in relevant studies. Taking the human–machine cooperation relationship as the moderating variable, this study estimates the influencing conditions of job demands on competency requirements, reveals the contingent factors existing in the relationship between the two, challenges the boundaries of traditional work relations, and develops the related research on the topic of the human–machine cooperation relationship in organizations.

### 5.2. Management Implications

The enlightenment arising from the findings presented in this article on management practice can be summarized in the following three ways:

First, managers should face the challenges that AI brings to enterprise employees. The application of AI technology in the workplace imposes more onerous requirements on employee knowledge and skills. It is the general trend for employees to improve their skills. At the initial stage of the application of AI, people and intelligent machines are in the running period. Employees need to spend considerable time and energy exploring skill-improvement paths to meet job demands, including allowing for their enthusiasm for learning to decline; therefore, enterprises should conduct targeted training on knowledge and skills related to AI for employees, shorten the running period between people and intelligent machines, reduce energy consumption, and realize the dynamic matching of employees' knowledge and skills with the requirements of new positions as soon as possible to stimulate employee learning.

Second, managers should strengthen the psychological counselling of employees and gradually adapt to the change in AI technology. In the application of AI, enterprises should enhance the psychological counselling and intervention of employees; they should help employees move from the initial rejection and resistance to AI to adaptation, acceptance, and recognition and embrace AI technology with a positive attitude. In addition, after the introduction of AI technology, enterprises should not only release workers from boring and repetitive work and improve labor productivity but also give them more meaning to complete high-level work with high complexity and high value.

Third, AI has made subversive changes in labor forms, methods, and processes. Corporate executives should create a culture of innovation in the context of human–machine cooperation, communicate with intelligent robots with an equal mindset, promote human–computer interaction, open new models of emotional and physical interaction, and enhance human–machine interdependence. Corporate executives should also guide employees to embrace new technologies of AI, look for opportunities to create new value, and master the skills required for the development of AI.

### 5.3. Limitations

The study has several limitations despite its valuable contributions to the literature. First, this study uses cross-sectional data, which are unable to reveal the process and mechanism underpinning the impact of job demands on employee learning. In the future, a longitudinal research design can be used to collect data at different time points to compensate for the lack of a causality test. Second, the application of AI technology affects not only job demands but also job characteristics, such as job autonomy and skill diversity. The impact of these job characteristics on employee learning warrants further investigation. Third, under the AI scenario, the human–machine cooperation relationship has not been accurately defined and measured yet, so further exploration is necessary.

### 5.4. Technical Implementation Challenges

There are also some technical implementation challenges. First, answers to the questionnaire are inevitably affected by human factors. Although the questionnaire was distributed at different times to reduce the influences of human factors, respondent cognition, and the situation when they completed the questionnaire would affect the answers to certain items. It is difficult for us to ascertain what the respondents think and do. Second, the questionnaire is not flexible. We have designed the answer scope for some questions in advance, which limits the respondents' answers and may omit more detailed information. For example, after the introduction of AI into the firm, changes in job demands affect employee learning, and the effects may be different among different environments or employees. Unfortunately, it is difficult to reflect them in the questionnaire. Therefore, a variety of methods were employed to verify the research questions in the future research, such as using experimental methods to validate the intuitive changes to employees after the introduction of AI into the firm or using big data statistical methods to determine the impact of job demands on employee learning.

## 6. Conclusion

This study explores the mechanism by which job demands affect employee learning in AI application scenarios. Specifically, job demands have a *U*-shaped curvilinear effect on employee learning. Furthermore, the human–machine cooperation relationship moderates the *U*-shaped curvilinear relationship between job demands and employee learning. Our conceptual model addresses the relationship between human–machine cooperation relationship and employee learning, and it opens many new fascinating lines of inquiry for future research. Moreover, our model provides theoretical suggestions for firms to improve learning enthusiasm through psychological counselling, vocational skills training, and others.

Employee learning plays an essential role in organizational development, particularly after the introduction of AI into the work field. Employee psychology and behavior are facing greater challenges, such as burnout at work, anxiety about unemployment, and resistance to AI. Given these challenges, more studies are needed in this stream of research.

## Figures and Tables

**Table 1:** Descriptive statistics.

Variables	Sample (*n* = 500)	Percentage (%)
Gender		
Male	286	57.2
Female	214	42.8

Age		
18–25 years	42	8.4
26–35 years	165	33
36–45 years	168	33.6
46–54 years	99	19.8
55 years and above	26	5.2

Education		
Secondary/high school	74	14.8
Undergraduate degree	352	70.4
Postgraduate degree and above	74	14.8

**Table 2:** Analysis of reliability and validity.

Variable	Combination reliability		Convergence reliability
	CR	Cronbach's *α*	AVE
Job demands	0.802	0.777	0.510
Human–machine cooperation relationship	0.706	0.700	0.491
Employee learning	0.786	0.635	0.479

**Table 3:** Confirmatory factor analysis results.

Model	(*χ*^2^/d*f*)	RMSEA	CFI	TLI
Three-factor model	2.604	0.058	0.914	0.906
Two-factor model	3.231	0.067	0.903	0.858
Single-factor model	3.923	0.077	0.871	0.813

Note. (*χ*^2^/d*f*) represents the goodness of fit index, if (*χ*^2^/d*f*) < 3, it shows the goodness of fit of the model; the root-mean-square error of approximation (RMSEA) denotes the index showing that the evaluation model does not fit, if RMSEA < 0.08, it shows the goodness of fit of the model; the comparative fit index (CFI) and Tucker–Lewis Index (TLI) represent a comparative fit index; if CFI > 0.9 and TLI > 0.9, respectively, it shows that the goodness of fit of the model is within an acceptable range.

**Table 4:** Analysis of descriptive statistics and correlation.

Variable	Mean	Standard deviation	1	2	3
Job demands	3.652	0.742	1		
Human–machine cooperation relationship	3.953	0.619	0.275	1	
Employee learning	4.177	0.420	0.253^∗∗^	0.335	1

*Note. N* = 500; ^∗∗^*p* < 0.01.

**Table 5:** Regression analysis results.

Variable	Learning			
	M1	M2	M3	M4
Gender	0.080^∗^	0.073^∗^	0.056^+^	0.051
Age	−0.014	−0.009	−0.01	−0.002
Education	−0.133^∗∗∗^	−0.122^∗∗∗^	−0.096^∗∗∗^	0.049^+^
Position	0.079^∗∗^	0.064^∗^	0.046^+^	−0.096^∗^
Job demands	0.134	−0.583^∗∗∗^	−0.544^∗∗∗^	−0.596
Job demands square		0.101^∗∗∗^	0.089^∗∗∗^	0.083
Human–machine cooperation relationship				0.123
Job demands × human–machine cooperation relationship				−0.037
Job demands square × human–machine cooperation relationship				0.095^∗^
*R* ^2^	0.116	0.145	0.266	0.302
△*R*^2^	—	0.029^∗∗^	0.121^∗∗∗^	0.036^∗∗^
*F*	12.909^∗∗∗^	13.944^∗∗∗^	25.422^∗∗∗^	21.162

*Note. N* = 500; ^+^*p* < 0.1, ^∗^*p* < 0.05, ^∗∗^*p* < 0.01, ^∗∗∗^*p* < 0.001.

**Table 6:** Simple estimates and significance of the slope of the moderating effect.

Dependent variable	Independent variable	Moderator	Simple estimate of the slope	Standard error	95% confidence interval	
					Lower	Higher
Employee learning	Job demands	High human–machine cooperation relationship	0.019^∗∗∗^	0.005	0.009	0.029
		Low human–machine cooperation relationship	0.013^∗^	0.005	0.003	0.023

*Note. N* = 500; ^∗^*p* < 0.05, ^∗∗∗^*p* < 0.001.

## Data Availability

The data used to support the findings of this study are available from the corresponding author upon request.
